# Influence of Low Protein Diet-Induced Fetal Growth Restriction on the Neuroplacental Corticosterone Axis in the Rat

**DOI:** 10.3389/fendo.2019.00124

**Published:** 2019-03-11

**Authors:** Marius Schmidt, Manfred Rauh, Matthias C. Schmid, Hanna Huebner, Matthias Ruebner, Rainer Wachtveitl, Nada Cordasic, Wolfgang Rascher, Carlos Menendez-Castro, Andrea Hartner, Fabian B. Fahlbusch

**Affiliations:** ^1^Department of Pediatrics and Adolescent Medicine, Friedrich-Alexander University Erlangen-Nuremberg, Erlangen, Germany; ^2^Institute of Medical Biometry, Informatics and Epidemiology, Faculty of Medicine, Rheinische Friedrich-Wilhelms-University, Bonn, Germany; ^3^Department of Gynaecology and Obstetrics/Comprehensive Cancer Center Erlangen-EMN, Friedrich-Alexander University Erlangen-Nuremberg, Erlangen, Germany

**Keywords:** IUGR intrauterine growth restriction, steroids, brain, placenta, Hsd11b2, rat model, low protein, neuroplacentology

## Abstract

**Objectives:** Placental steroid metabolism is linked to the fetal hypothalamus-pituitary-adrenal axis. Intrauterine growth restriction (IUGR) might alter this cross-talk and lead to maternal stress, in turn contributing to the pathogenesis of anxiety-related disorders of the offspring, which might be mediated by fetal overexposure to, or a reduced local enzymatic protection against maternal glucocorticoids. So far, direct evidence of altered levels of circulating/local glucocorticoids is scarce. Liquid chromatography tandem-mass spectrometry (LC-MS/MS) allows quantitative endocrine assessment of blood and tissue. Using a rat model of maternal protein restriction (low protein [LP] vs. normal protein [NP]) to induce IUGR, we analyzed fetal and maternal steroid levels via LC-MS/MS along with the local expression of 11beta-hydroxysteroid-dehydrogenase (*Hsd11b*).

**Methods:** Pregnant Wistar dams were fed a low protein (8%, LP; IUGR) or an isocaloric normal protein diet (17%, NP; controls). At E18.5, the expression of *Hsd11b1* and *2* was determined by RT-PCR in fetal placenta and brain. Steroid profiling of maternal and fetal whole blood, fetal brain, and placenta was performed via LC-MS/MS.

**Results:** In animals with LP-induced reduced body (*p* < 0.001) and placental weights (*p* < 0.05) we did not observe any difference in the expressional *Hsd11b1/2*-ratio in brain or placenta. Moreover, LP diet did not alter corticosterone (Cort) or 11-dehydrocorticosterone (DH-Cort) levels in dams, while fetal whole blood levels of Cort were significantly lower in the LP group (*p* < 0.001) and concomitantly in LP brain (*p* = 0.003) and LP placenta (*p* = 0.002). Maternal and fetal progesterone levels (whole blood and tissue) were not influenced by LP diet.

**Conclusion:** Various rat models of intrauterine stress show profound alterations in placental Hsd11b2 gatekeeper function and fetal overexposure to corticosterone. In contrast, LP diet in our model induced IUGR without altering maternal steroid levels or placental enzymatic glucocorticoid barrier function. In fact, IUGR offspring showed significantly reduced levels of circulating and local corticosterone. Thus, our LP model might not represent a genuine model of intrauterine stress. Hypothetically, the observed changes might reflect a fetal attempt to maintain anabolic conditions in the light of protein restriction to sustain regular brain development. This may contribute to fetal origins of later neurodevelopmental sequelae.

## Introduction

The association of intrauterine growth restriction (IUGR) with the development of metabolic disorders [i.e., type 2 diabetes, hyperlipidemia (1)] and cardiac disease [i.e., arterial hypertension, cardiovascular disease (2)] in later life is well-recognized based on several animal (3, 4) and human (5) studies. Barker introduced the hypothesis of “fetal programming,” postulating that an adverse intrauterine environment affects growth and differentiation of the fetus and subsequently contributes to the development of the above sequelae later in life (6).

The mechanistic basis of “developmental origins of health and disease” (DOHaD) are subject of ongoing research (7). A possible mechanism that links maternal gestational adversity to long-term health outcomes in offspring is the diaplacental transfer of glucocorticoids (8): during pregnancy the placental enzyme 11beta-hydroxysteroid dehydrogenase 2 (Hsd11b2) catalyses the oxidation of cortisol to cortisone [predominant in humans (9)] and of corticosterone (Cort) to 11-dehydrocorticosterone [DH-Cort, predominant in rats (9)], thereby inactivating these glucocorticoids. There is evidence that low birth weight might be associated with a reduced placental HSD11b2 activity in both humans (10) and rodents (11–13) contributing to fetal hypercortisolism and programming of hypertension and metabolic syndrome in adult rat offspring.

Beyond these major schemes of fetal programming, a potential negative influence of poor fetal growth on brain development with anxiety-related sequelae is being critically discussed (14). In human pregnancy, heightened maternal anxiety with endogenous elevation of glucocorticoids might predispose the offspring for schizophrenia (15), hyperactive disorder (16) and/or impaired emotional and cognitive development (17). It has been shown that maternal anxiety is negatively correlated with placental *HSD11b2* mRNA expression (18). Additionally, a reduction of placental HSD11b2 seems to be associated with IUGR (19). We have also found that the *HSD11b2* mRNA expression is negatively correlated with postnatal catch-up growth in IUGR (10).

There is evidence that iatrogenic administration of exogenous glucocorticoids for lung maturation might exert negative fetal effects: A single course of antenatal betamethasone (not metabolized by HSD11b2) treatment before 34 + 0 weeks of gestation seems to be associated with an impairment of cognitive ability in treated infants (20, 21) and a reduced head circumference in females (22) at term. Moreover, betamethasone treatment (23) and inhibition of HSD11b2 activity (24) was associated with a certain impairment of hypothalamic-pituitary-adrenal (HPA)-axis function in humans.

Similar to humans, fetal exposure of rats to gestational stress (e.g., via inhibition of Hsd11b2 or treatment with glucocorticoids) seemed to induce low birth weight and trigger processes in the HPA-axis with negative effects on postnatal neurodevelopment. Affected rats showed increased stress responsivity, anxiety-like behavior and altered social interaction postnatally (25–28). Experiments with Hsd11b2 null mice are indicative of direct fetal programming effects on animal behavior via endogenous glucocorticoids (29).

Various non-surgical rat models for the induction of gestational maternal adversity exist (28). In general, maternal stress is either directly [e.g., chronic restraint stress (30)] or indirectly [e.g., nutritional restriction (13, 31)], induced during various stages of gestation. Maternal glucocorticoids are deemed to be among the main effectors of fetal programming in these animals (32), as prenatal effects can be averted via adrenalectomy of the dams (33). Noteworthy, the glucocorticoid system is known to be tightly interlinked with other essential regulators of fetal neurodevelopment, such as the neuroplacental serotonin (5-hydroxytryptamine; 5-HT) axis (34, 35). Perturbations of the fetal serotonin system during early development by prenatal maternal stress have been linked to processes of fetal programming of psychiatric disorders in later life (35).

In our study we investigated the maternal and fetal (E18.5) HPA-axis in an established nutritionally-induced IUGR model (36), using a novel methodological approach. So far, endocrine changes in such models were mainly studied using multiple ELISA measurements of single hormones in serum or RT-PCR of *Hsd11b2* in target tissues (i.e., placenta and brain) (11, 12, 22, 37–40). We have recently established a liquid chromatography tandem mass-spectrometry method (LC-MS/MS) that allows for the detection of multiple glucocorticoids in a single tissue/serum probe (9, 41), minimizing tissue-matrix interactions (42, 43). The combination of LC-MS/MS with volumetric adhesive microsampling devices enables individual sampling without the need for pooling of fetal samples (44). We set out to determine the steroid profile in maternal and fetal circulation, along with placental and brain steroid profiles, in IUGR vs. control rats and to compare these findings to *Hsd11b2* mRNA expression in these tissues. We were especially interested to identify steroid profiles characteristic for IUGR. In the face of potential IUGR-related neurologic sequelae and the role of placental Hsd11b2, we aimed to provide evidence of a neuroplacental cross-talk on the glucocorticoid level in these animals.

## Methods

### Animals and Diets

Animal procedures were carried out as previously described (36). This study was carried out in accordance with the recommendations of the NIH *Guide for the Care and Use of Laboratory Animals* and the *EU Directive 2010/63/EU*. All procedures and protocols were governmentally approved by the corresponding board (Regierung von Mittelfranken, AZ #54-2531.31-31/09). Wistar rats were ordered from Charles River (Sulzfeld, Germany). Virgin female rats (240–260 g) were housed individually and maintained at 22°C on a 12 h light–dark cycle. After mating, pregnancy was confirmed via vaginal plug formation (day 1 of gestation). The use of our alimentary IUGR model for the analysis of postnatal sequelae of fetal programming has been previously described by us (3, 36, 45, 46). In short, 12 dams were randomly assigned to two groups receiving semi-purified diets (Altromin Spezialfutter GmbH & Co. KG, Lage, Germany) of either low protein diet (LP group, 25 g/d of Altromin C1003, 8.1% protein, 13% fat, 78% carbohydrates [2% monosaccharides, 18% disaccharides, 49% polysaccharides] or an isocaloric diet of normal protein content (NP group, 25 g/d of Altromin C1000, 17.3% protein, 13% fat, 67% carbohydrates [10% disaccharides, 47% polysaccharides], and were weighed daily. Animal characteristics are displayed in Table 1.

**Table 1:** Rat auxologic data.

**Weight (g)**	**NP**	**LP**	***p*-value**
Litter (*n*)	4	5	
Fetus	1.38 ± 0.09	0.84 ± 0.06	<0.001^#^
Brain	0.08 ± 0.01	0.08 ± 0.05	ns^§^
Placenta	0.34 ± 0.04	0.29 ± 0.02	<0.050^#^
Placenta/Fetus	0.25 ± 0.02	0.35 ± 0.03	<0.001^#^
Litter size	12.75 ± 2.22	14.20 ± 2.59	ns^#^

(#) Welch's t-test,

(§) *Mann–Whitney U-test*.

Additionally, a second set of four dams (conversion set) was mated and received NP diet *ad libitum*. Blood samples of these animals and the respective fetuses were used to establish a conversion factor for volumetric adhesive microsampling (VAMS) to EDTA-blood in order to improve comparability of data with findings from the literature (Supplementary Table S1). Moreover, LC-MS/MS pretesting of placental tissue and brain in these animals helped to optimize run settings for steroid and CRH detection.

### Sample and Data Collection

At E18.5 fetuses were obtained via cesarean section under isoflurane anesthesia. Starting out with six dams per group, one dam of the NP group (NP) had to be excluded as litter size (<10 fetuses) was extremely low. Additionally, two dams (one NP and LP, each) did not follow their expected growth trajectories and were subsequently excluded by the veterinarian due to suspected health issues. For our final analysis six fetuses (*n* = 3 females, *n* = 3 males each) per dam (*n* = 4 NP, *n* = 5 LP) were chosen based on their matching positions in the proximal uterine horns. Fetuses were sacrificed via decapitation, followed by individual Mitra™ (Neoteryx, LLC, Torrance, CA, USA) VAMS of mixed arterio-venous blood from the trunk's cutting surface, as described (44). Brain and placenta samples were instantly snap-frozen in liquid nitrogen and stored at −80°C. Fetal tail-tip biopsies were used for sex determination. We did not perform fetal saline-perfusion prior to tissue collection, as this procedure has been shown to interfere with cerebral tissue steroid detection (47). Subsequently, dams were sacrificed via aortic transection. Arterial blood was drawn via syringe and collected in K3 EDTA blood collection tubes (Sarstedt, Nümbrecht, Germany). Additionally, whole blood samples were collected via VAMS.

In the conversion set of animals (see above), trunk blood of fetuses from one litter was pooled in EDTA-tubes to facilitate the measurement of a maximum number of steroids for the purpose of converting VAMS steroid measurements into EDTA standard values (Supplementary Table S1).

### RNA Extraction, RT-PCR, and Real-Time Quantitative PCR

For measurement of *Hsd11b1* and *2*, RNA was extracted from fetal placenta and brain using peqGOLD TriFast™, according to the manufacturer's instruction (VWR International GmbH, Darmstadt, Germany). RNA concentration was quantified via NanoDrop ND1000 spectrophotometry (Peqlab) and adjusted to 1 ng/ml. After DNase treatment, RNA was transcribed into cDNA using the QuantiTect Reverse Transcription Kit (Qiagen, Hilden, Germany) and random hexamers as primers. Reactions without Quantiscript Reverse Transcriptase served as negative controls. Quantitative real-time PCR was performed with SYBR Green (Applied Biosystems, Darmstadt, Germany; ThermoFisher Scientific) using the StepOnePlus Real-Time PCR System (Applied Biosystems). Samples were run in duplicates and mRNA levels were normalized to the housekeeping gene *18S* rRNA. SYBR-Green based real-time PCR results were compared between groups using the 2^−ΔΔ*CT*^-method. Primers sequences were as following (5′-3′): *r18s* forward (fw) TTGATTAAGTCCCTGCCCTTTGT, *r18s* reverse (rev) CGATCCGAGGGCCTCACTA; *Hsd11b1* fw TAGACACAGAAACAGCTTTG, *Hsd11b1* rev AATTCCATGATCCTCCTTCC; *Hsd11b2* fw CAGGAGACATGCCATACC, *Hsd11b2* rev GATGATGCTGACCTTGATAC.

Sex verification was carried out via sex-determining region Y (*Sry*) gene PCR (45). DNA from tail-tip biopsies was extracted using the MyTaq™ Extract-PCR Kit (Bioline, London, UK). Sex determination was performed via commercially available PCR *Sry* assay (Rn04224592; ThermoScientific, Waltham, USA) with *18S* rRNA as housekeeping gene. PCR conditions were adopted from the manufacturer's protocol (ThermoFisher). For male gender a cut-off <23 cycles was chosen, while the threshold for female sex was >29 cycles. When verification was needed, we repeated the measurement with corresponding liver samples.

### Liquid Chromatography—Tandem Mass Spectrometry (LC-MS/MS)

We have previously established and validated LC-MS/MS methods for the determination of steroids in human placenta (41) and amniotic fluid (48), as well as in rat placenta and brain (9). We determined corticosterone (Cort), 11-dehydrocorticosterone (DH-Cort), 11-deoxycorticosterone (DOC), progesterone, androstenedione, and testosterone in maternal and fetal whole blood, fetal brain, and placenta. While others were able to detect testosterone in pooled fetal plasma on E19 by RIA (49), androstenedione and testosterone were below the detection limit in whole blood (data not shown), due to individual sampling. LC-MS/MS allows for the combined quantification of corticotropin-releasing hormone (CRH) and steroids. As CRH is not present in rat placenta (9), our study involves its determination in fetal brain only. The following adjustments were made to the methods above: We used 1 ml of ethanol solvent containing protease inhibitor cocktail (Aprotinin 2.0 μl/ml, Pepstatin 6.7 μl/ml and Leupeptin 10.0 μl/ml, all from Carl Roth, Karlsruhe, Germany) per 150 mg tissue for homogenization and waived ultrasonification based on tissue softness. The reduced fetal organ size allowed for the reduction of the amount of resuspended tissue homogenate to 1/10th. Thus, 30 μl of homogenized supernatant were added to 90 μl methanol (MeOH) containing 1% formic acid (FA) for LC-MS/MS sample preparation. Thereby, we could maintain our established sample to solution ratio of 1:3. For steroid profiling, sample protein precipitation was adjusted to the initial reduction of homogenate quantity, such that 50 μl of the above methanol/FA supernatant was added to 50 μl MeOH/zinc sulfate (50 g/L, 1/1 v/v), while the addition of 100 μl internal standard remained unmodified (41). The concentration of standards dilution series encompassed a range of 1.0−250.0 ng/ml. CRH quantification was performed with the remaining homogenate *in toto*. We added MeOH/FA (99/1 v/v) containing 0.24 μg/ml bovine CRH as internal standard (IS, Bachem AG, Bubendorf, Switzerland) relative to 1/3rd of the homogenate volume. Serial standard dilutions of CRH with equivalent IS-ratio covered a detection range of 0.1–20.0 ng/ml. LC-MS/MS measurements of steroids in VAMS of fetal whole blood via MITRA capillary blood collection device (Neoteryx LLC, Torrance, CA, USA) has been previously established and validated by us (44). The final fetal and maternal conversion factors from VAMS whole blood to EDTA blood are given in Supplementary Table S1.

### Statistical Analysis

Data processing and imaging was performed with Microsoft Office 2016 (Microsoft, Redmond, WA, USA) and Adobe Photoshop CS6 (Adobe Systems, San José, CA, USA). For statistical analysis we used GraphPad PRISM Version 7.0 (GraphPad Software, La Jolla, CA, USA). For every litter, the mean levels of the respective individual fetal hormones in tissue and blood were calculated. Outliers of all laboratory parameters and respective ratios within litters and groups were corrected by using the PRISM “robust regression and outlier removal” (ROUT) method (Q = 1%, equivalent to a false discovery rate of 1%), as described by Motulsky and Brown (50) and Hughes and Hekimi (51). Excluded data points (*n* = 6) are given in Supplementary Table S2 and were not included in the calculation of the mean per litter. Subsequently, the means per litter (*n* = 4 for the NP group, *n* = 5 for the LP group) were subjected to further statistical analysis. Before performing groupwise comparisons, we checked the normality assumption of the mean values by carrying out a Shapiro Wilk test in each experimental group, followed by a Bonferroni correction of the resulting *p*-values (one per experimental group). The null hypothesis of normality in both groups was rejected if at least one Bonferroni-corrected Shapiro Wilk *p* < 0.05. When normal distribution was present, Welch's *t*-test was performed, otherwise a Mann–Whitney *U*-test was executed. For sex-specific subgroup comparison between NP and LP groups, we used two-way analysis of variance (two-way ANOVA with Sidak's multiple comparison analysis). The normality assumption for the ANOVA residuals was verified using normal quantile-quantile (QQ) plots. All data in this manuscript represent mean ± standard deviation (SD) regardless of their normality, facilitating comparative overview. A *p* < 0.05 was considered statistically significant.

## Results

### Auxologic Data

We did not observe significant differences in litter size in our LP group secondary to their dietary restriction (Table 1). The fetal and placental weights were both significantly lower in the LP group compared to the NP group (*p* < 0.001 and *p* < 0.05, respectively, Table 1), while brain weight did not differ (Table 1). The ratio of placenta-to-body weight was significantly increased in the LP group (*p* < 0.001, Table 1). Thus, while LP diet negatively affected both body and placental weight, its influence on fetal body weight appeared to be more prominent (Table 1).

### Quantitative Real-Time PCR (qRT-PCR)

**Figure 1:**
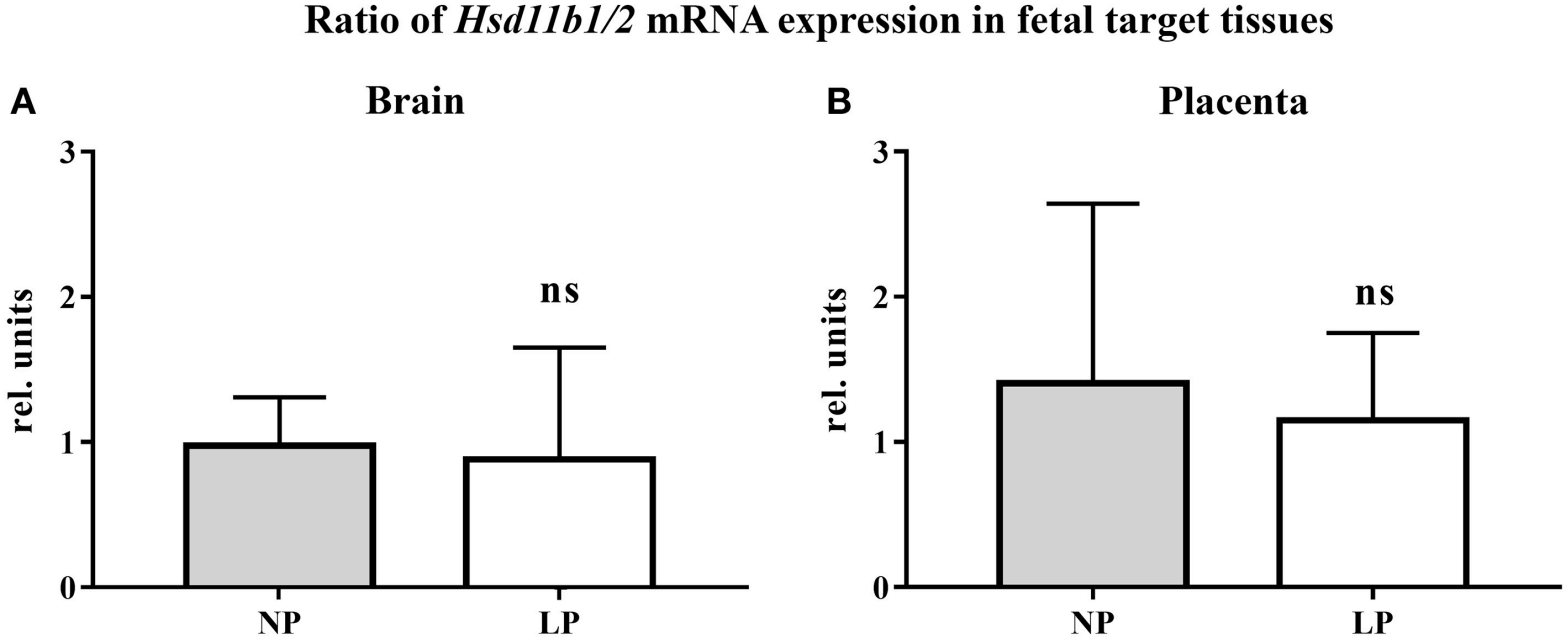
Ratio of *Hsd11b1/2* mRNA expression (qRT-PCR, relative units) in fetal target tissues. **(A)** Fetal rat brain, **(B)** Rat placenta. Legend: Gray column represents NP group (*n* = 4), white column represents LP group (*n* = 5). Legend: rel. units, relative units; ns, not significant.

The results of our qRT-PCR of fetal brain and placental tissue are listed in Table 2. We did not detect significant differences in *Hsd11b1* and *2* mRNA expressions between the NP and LP groups in both brain and placenta. Moreover, the ratio of *Hsd11b1* to *Hsd11b2* was not significantly influenced by LP diet in brain (Figure 1A) and placenta (Figure 1B). Sex-specific analysis of *Hsd11b1/2*-ratio between NP and LP groups did not reveal significant differences (data not shown).

**Table 2:** qRT-PCR results.

		**NP**	**LP**	***p*-value**
Brain	*n*	4	5	
	Fold change (*Hsd11b1*)	1.00 ± 0.69	0.89 ± 0.94	ns^#^
	Fold change (*Hsd11b2*)	1.00 ± 0.51	1.11 ± 0.53	ns^#^
	Ratio	0.98 ± 0.33	0.88 ± 0.77	ns^#^
Placenta	Fold change (*Hsd11b1*)	1.00 ± 0.92	1.96 ± 0.84	ns^#^
	Fold change (*Hsd11b2*)	1.00 ± 0.82	1.84 ± 0.55	ns^#^
	Ratio	1.40 ± 1.24	1.15 ± 0.60	ns^#^

(#) *Welch's t-test*.

### Steroid Analysis via LC-MS/MS in Maternal and Fetal Whole Blood

**Figure 2:**
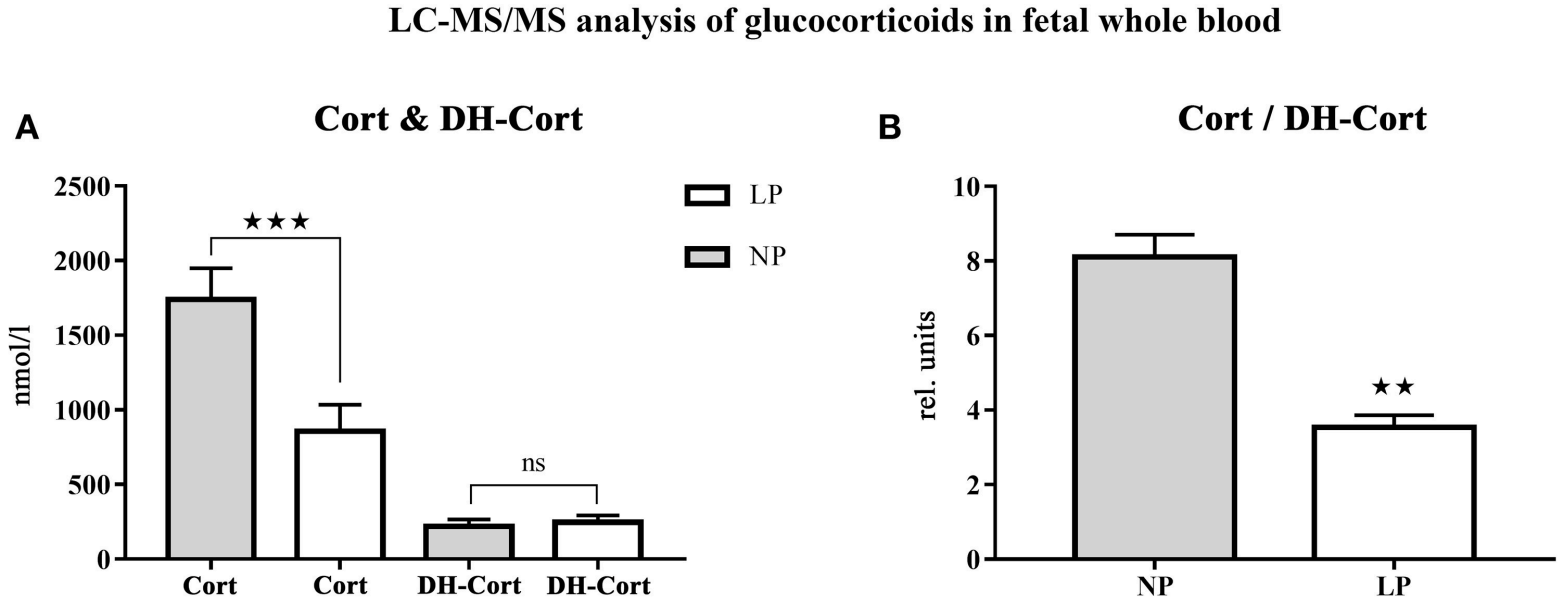
LC-MS/MS analysis of circulating glucocorticoid levels in fetal whole blood. **(A)** Differences of circulating corticosterone (Cort) and 11-dehydrocorticosterone (DH-Cort) levels of fetal NP (*n* = 4) and LP (*n* = 5) whole blood (pmol/g). **(B)** Differences of Cort/DH-Cort -ratio in whole blood. Legend: Gray column represents NP group (*n* = 4), white column represents LP group (*n* = 5), rel. units, relative units; ns, not significant; ^**^p < 0.01 and ^***^p < 0.001.

Steroid profiles of maternal, as well as of fetal whole blood can be found in Tables 3, 4, respectively. NP and LP dams showed similar profiles of circulating steroids and Cort/DH-Cort-ratios (Table 3). In LP fetuses circulating levels of corticosterone were significantly reduced by 50.78% (*p* < 0.001, Figure 2A), while significant group differences regarding circulating levels of 11-dehydrocorticosterone were not detected (Table 4, Figure 2A). The ratio of Cort/DH-Cort was significantly reduced in LP rat fetuses (*p* = 0.001, Table 4, Figure 2B). As the pools of fetal and maternal glucocorticoids are diaplacentally linked (52, 53), we determined the feto-maternal Cort/DH-Cort-ratio (i.e., normalization of fetal to maternal steroid levels). No significant alteration of fetal Cort/DH-Cort-ratio was found in the LP group compared to NP fetuses (data not shown). Moreover, we found a significant reduction in circulating DOC levels in the LP group (*p* = 0.028, Table 4), while progesterone levels were not significantly different.

**Table 3:** Steroid analysis of maternal whole blood.

**Steroids in maternal whole blood (nmol/l)**	**NP**	**LP**	***p*-value**
*n*	4	5	
Corticosterone (Cort)	1333.00 ± 343.30	1191.00 ± 289.20	ns^§^
Dehydrocorticosterone (DH-Cort)	91.21 ± 25.23	89.12 ± 34.19	ns^#^
Ratio (Cort/DH-Cort)	15.32 ± 5.51	14.37 ± 3.71	ns^#^
Deoxycorticosterone (DOC)	27.63 ± 17.12	14.63 ± 6.49	ns^#^
Progesterone	761.40 ± 466.50	430.00 ± 177.90	ns^§^
Testosterone	1.68 ± 0.89	2.21 ± 0.47	ns^#^

(#) Welch's t-test,

(§) *Mann–Whitney U-test*.

**Table 4:** Steroid analysis of fetal whole blood.

**Steroids in fetal whole blood (nmol/l)**	**NP**	**LP**	***p*-value**
*n*	4	5	
Corticosterone (Cort)	1740.00 ± 209.90	856.50 ± 177.10	<0.001^#^
Dehydrocorticosterone (DH-Cort)	218.80 ± 47.30	246.10 ± 46.12	ns^#^
Ratio (Cort/DH-Cort)	8.10 ± 1.20	3.54 ± 0.73	0.001^#^
Deoxycorticosterone (DOC)	72.23 ± 16.56	42.54 ± 9.41	0.028^#^
Progesterone	31.52 ± 4.94	29.38 ± 7.09	ns^#^

(#) *Welch's t-test*.

Subsequently, we examined fetal steroid profiles with regard to sex (Table 5). This subgroup analysis revealed no sex-specific differences between NP and LP groups, with regard to circulating steroids.

**Table 5:** Sex-specific steroid analysis of fetal whole blood.

	**Steroids in fetal whole blood (nmol/l)**	**NP**	**LP**	***p*-value**
	*n*	4	5	
Male	Corticosterone (Cort)	1803.00 ± 191.20	838.50 ± 152.30	<0.001^‡^
	Dehydrocorticosterone (DH-Cort)	210.40 ± 56.29	238.50 ± 67.45	ns^‡^
	Ratio (Cort/DH-Cort)	8.95 ± 2.22	3.71 ± 1.07	<0.001^‡^
	Deoxycorticosterone (DOC)	73.71 ± 17.00	42.06 ± 14.24	0.014^‡^
	Progesterone	33.02 ± 4.31	27.27 ± 13.01	ns^‡^
Female	Corticosterone (Cort)	1677.00 ± 239.00	874.40 ± 218.40	<0.001^‡^
	Dehydrocorticosterone (DH-Cort)	227.20 ± 47.31	253.70 ± 41.84	ns^‡^
	Ratio (Cort/DH-Cort)	7.48 ± 0.78	3.49 ± 0.90	0.001^‡^
	Deoxycorticosterone (DOC)	70.75 ± 16.61	43.02 ± 12.40	0.030^‡^
	Progesterone	30.01 ± 7.05	31.49 ± 17.89	ns^‡^

(‡) *Two-way Anova*.

### Steroid Analysis via LC-MS/MS in Fetal Tissue

**Figure 3:**
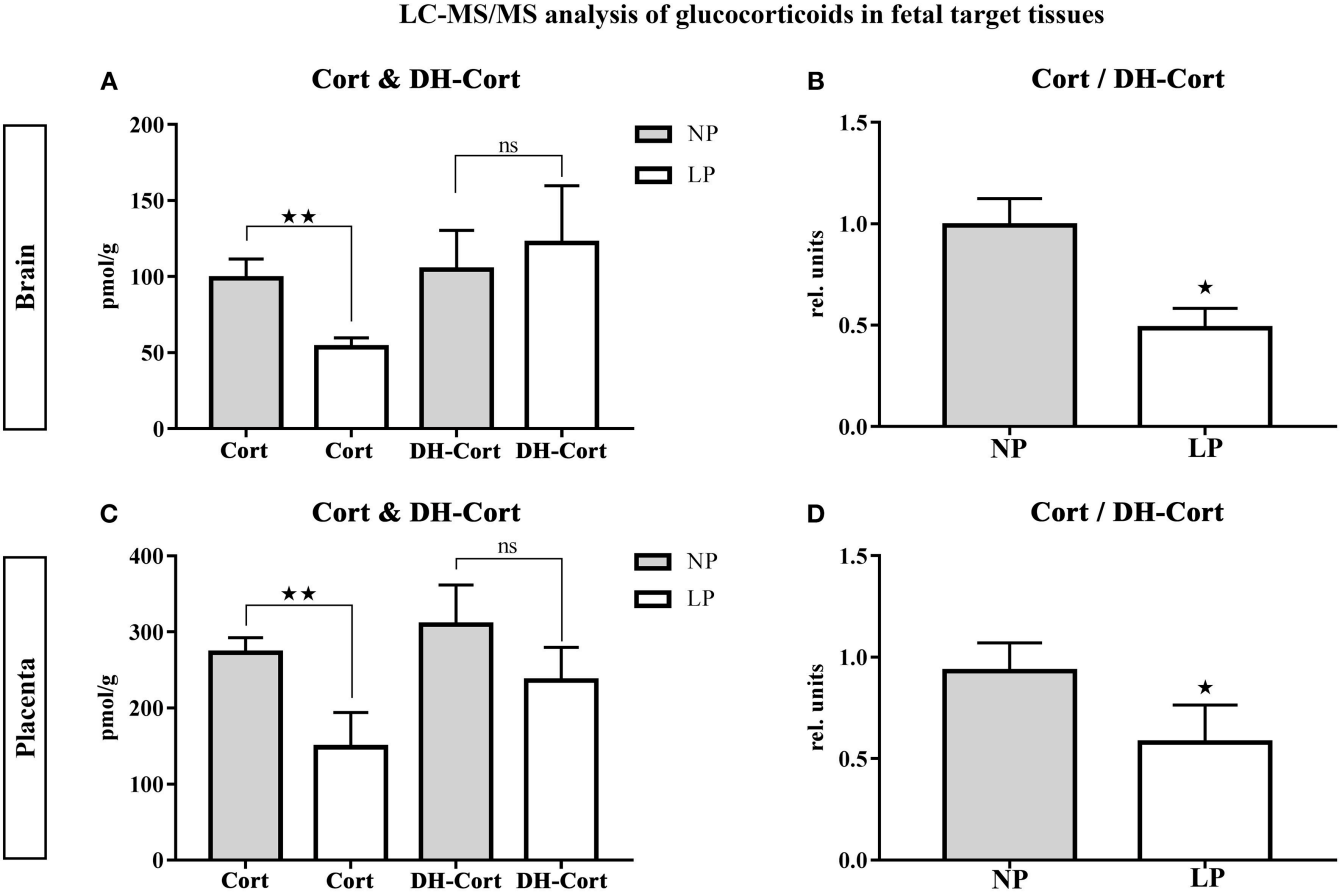
LC-MS/MS analysis of local glucocorticoid levels in fetal target tissues. Results of fetal brain are shown in **(A,B)**, results of placenta are shown in **(C,D)**. **(A)** Differences of corticosterone (Cort) and 11-dehydrocorticosterone (DH-Cort) levels in fetal brains of NP (*n* = 4) and LP (*n* = 5) groups (pmol/g). **(B)** Differences of Cort/DH-Cort -ratio in fetal brain (relative units). **(C)** Differences of corticosterone (Cort) and 11-dehydrocorticosterone (DH-Cort) levels in placenta of NP (*n* = 4) and LP (*n* = 5) groups (pmol/g). **(D)** Differences of Cort/DH-Cort-ratio in placenta of NP (*n* = 4) and LP (*n* = 5) groups (relative units). Legend: Gray column represents NP group (*n* = 4), white column represents LP (*n* = 5) group, rel. units, relative units; ns, not significant; ^*^p < 0.05, ^**^p < 0.01.

Brain and placental tissue steroid profiles are listed in Tables 6, 7, respectively. LP diet significantly lowered local corticosterone levels of both brain and placenta by 45.75% (*p* = 0.003, Figure 3A) and 45.56% (*p* = 0.002, Figure 3C), respectively. In both brain and placenta, levels of 11-dehydrocorticosterone were not significantly altered compared to the NP groups (Table 6, Figure 3A; Table 7, Figure 3C, respectively). In the LP group the local Cort/DH-Cort-ratio was significantly lower in fetal brain (*p* = 0.016; Figure 3B; Table 6) and placenta (*p* = 0.014; Figure 3D; Table 7) when compared to NP. Furthermore, local DOC levels were significantly reduced in brain (*p* = 0.029, Table 6) and placenta (*p* = 0.036, Table 7) of LP fetuses. No significant differences of local progesterone in brain and placenta were observed.

**Table 6:** Steroid and CRH analysis of fetal brain.

**Steroids and CRH in fetal brain (pmol/g)**	**NP**	**LP**	***p*-value**
*n*	4	5	
Corticosterone (Cort)	98.86 ± 12.77	53.63 ± 6.09	0.003^#^
Dehydrocorticosterone (DH-Cort)	104.50 ± 25.86	122.10 ± 37.66	ns^#^
Ratio (Cort/DH-Cort)	0.99 ± 0.13	0.48 ± 0.10	0.016^§^
Deoxycorticosterone (DOC)	13.05 ± 2.67	7.68 ± 3.19	0.029^#^
Progesterone	47.64 ± 7.81	37.86 ± 12.29	ns^#^
Androstenedione	3.21 ± 0.48	2.35 ± 0.70	ns^#^
Testosterone	1.87 ± 0.49	1.02 ± 0.81	ns^#^
CRH (ng/g)	1.50 ± 0.87	0.68 ± 0.31	ns (0.156)^#^

(#) Welch's t-test,

(§) *Mann–Whitney U-test*.

**Table 7:** Steroid analysis of placenta.

**Steroids in fetal placenta (pmol/g)**	**NP**	**LP**	***p*-value**
*n*	4	5	
Corticosterone (Cort)	272.80 ± 19.63	148.50 ± 45.76	0.002^#^
Dehydrocorticosterone (DH-Cort)	309.80 ± 52.00	236.20 ± 43.48	ns^#^
Ratio (Cort/DH-Cort)	0.93 ± 0.14	0.58 ± 0.18	0.014^#^
Deoxycorticosterone (DOC)	17.56 ± 4.33	10.17 ± 1.65	0.036^#^
Progesterone	44.29 ± 12.86	45.89 ± 8.83	ns^#^
Androstenedione	7.94 ± 1.65	8.58 ± 2.24	ns^#^
Testosterone	1.09 ± 0.43	0.83 ± 0.25	ns^#^

(#) *Welch's t-test*.

**Figure 4:**
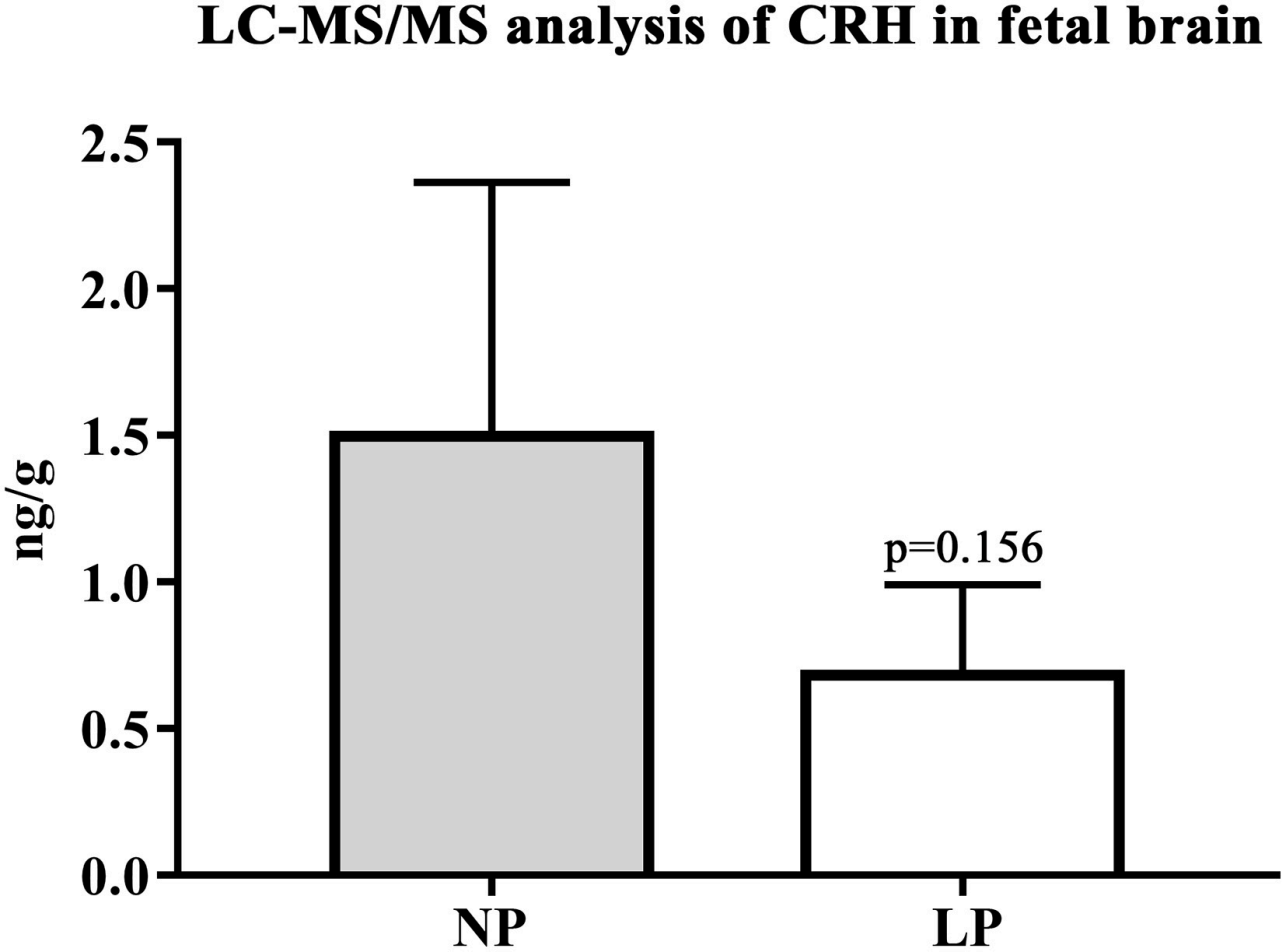
LC-MS/MS analysis of local CRH levels in fetal brain. Differences of CRH of NP (*n* = 4) and LP (*n* = 5) group in brain given in ng/g. Legend: Gray column represents NP (*n* = 4) group, white column represents LP (*n* = 5) group.

In the brain, LP diet significantly reduced local levels of androstenedione and testosterone in males only (*p* = 0.011, *p* = 0.025, respectively; Supplementary Table S3a). In contrast to humans (54), the rat placenta synthesizes significant amounts of androgens (androstenedione and testosterone) (55). In our model, local androgen levels of placenta did not significantly differ between NP and LP groups (Table 7), even when sex was regarded (Supplementary Table S3b). CRH levels were at the detection limit in fetal brain. Subsequently, we were only able to observe a trend to lower CRH in the brain of LP rats (*p* = 0.156, Table 6, Figure 4). CRH levels in the fetal brain did not show sex-specific differences between NP and LP groups (Supplementary Table S3a).

Further examining sex-specific local tissue steroid profiles of brain and placenta between NP and LP groups (Supplementary Table S2), we found that local levels of corticosterone in fetal brain and placenta were significantly diminished in both male and female LP rats (Supplementary Tables S3a,b, respectively). Furthermore, the sex-specific Cort/DH-Cort-ratio in these organs remained significantly reduced (Supplementary Table S3). However, the influence of LP diet on the Cort/DH-Cort-ratio reduction in the female placenta was only a trend (*p* = 0.086, Supplementary Table S3b). In the male brain, no difference between NP and LP groups was observed for DOC, while DOC was significantly reduced in female brain (*p* = 0.032, Supplementary Table S3a) and in the placenta of both sexes.

### Normalization of Tissue LC-MS/MS Results to Body Size

Glucocorticoids and mineralocorticoids are known to significantly influence brain and body weight in rats, respectively (56). As the LP group is characterized by a significant reduction of both body and placental weight (Table 1), we normalized local corticosterone, 11-dehydrocorticosterone and deoxycorticosterone levels of brain and placenta in both groups to their respective organ-to-body weight ratio (i.e., steroid x organ weight/body weight, data not shown). Cort/DH-Cort-ratio in brain and placenta of LP fetuses remained unaffected, i.e., significantly reduced (*p* = 0.016 and *p* = 0.014, respectively). Moreover, levels of placental corticosterone were significantly reduced (*p* = 0.043). No significance was obtained for deoxycorticosterone. While these results seemed largely independent of sex, the reduction of Cort/DH-Cort-ratio of female placenta remained a trend (*p* = 0.086, data not shown).

### Analysis of Steroid Levels Relative to Progesterone

In the rat, progesterone serves as a precursor for mineralocorticoids (DOC) and glucocorticoids (57). Thus, we analyzed the ratio of progesterone to DOC and corticosterone. The results are given in Supplementary Table S4. In general, ratios of progesterone to DOC and to corticosterone were increased in all LP specimens tested. However, significance was only reached for progesterone/corticosterone-ratio in whole blood (*p* = 0.026, Supplementary Table S4) and progesterone/DOC-ratio in placenta (*p* = 0.003, respectively; Supplementary Table S4).

## Discussion

Our study analyzed steroid profiles via LC-MS/MS in fetal and maternal whole blood and the corresponding fetal placentas and brains in an established rat model of IUGR by maternal protein restriction. We found that LP nutrition did not alter maternal steroid profiles at E18.5. Furthermore, LP diet did not induce fetal hypercortisolism in our model. Instead, levels of circulating corticosterone were lower in LP litters along with a significant reduction in Cort/DH-Cort-ratio. Circulating DOC levels were reduced in the LP group, while progesterone levels remained constant. These results were independent of sex. Similar to our observation in whole blood, we also found a reduction of fetal corticosterone and Cort/DH-Cort-ratio in our analysis of LP brain and placental steroid profiles.

### Effects of Low Protein Diet on Steroid Profiles and CRH of the Fetal Brain

The Cort/DH-Cort-ratio in the brain was largely independent of sex in NP and LP groups. While normalization to organ-to-body weight ratio did not affect Cort/DH-Cort-ratio, the observed reduction in DOC tissue levels seemed secondary to the reduced LP weight development (56). It has been shown by others (56) that levels of corticosterone inversely correlate with brain mass in the rat. Also, there is evidence that administration of antenatal betamethasone might be associated with a reduction in head circumference (22) at term and subsequent impairment of cognitive function in treated infants (20, 21). Thus, a reduced Cort/DH-Cort-ratio in our model might argue for a brain sparing effect [reviewed by Miller et al. (58)] in our LP group, as brain weight was not significantly different when compared to NP animals. Brain sparing is associated with asymmetric fetal growth due to increased allocation of nutrients, oxygen, as well as fetal cardiac output to the brain in comparison to other organ systems (58). While this effect promotes survival, it is now apparent that brain sparing might negatively affect brain development in IUGR fetuses (58, 59). Fetal adaptations to adverse intrauterine environment may contribute to an increased postnatal vulnerability for neurodevelopmental and behavioral problems (58, 59). While most animal studies [reviewed by Miller et al. (58)] examined brain sparing effects in rat models of hypoxia or ligation of the uterine artery, García-Contreras et al. (60) examined brain sparing in a malnourished piglet model. They were able to show that nutritional shortage was able to induce local changes in neurotransmitter levels (catecholamines and indoleamines) in swine fetuses, dependent of their sex (60). These transmitters are thought to be related to important cognitive functions (e.g., learning, memory, reward-motivated behavior and stress) (61). To our knowledge, such postnatal behavioral sequelae and neuroendocrine effects have not been studied in our rat model so far. Thus, the potential presence of brain sparing in our LP animals requires future investigation. In line with the observation of sex-specific influences of IUGR on neurotransmitters in swine fetuses (60), we observed differences in brain androgen metabolism (significant reduction of androstenedione and testosterone levels) in our LP male rat fetuses only. These effects might point to brain-specific alterations in local *de novo* synthesis (62) of androgens, as placental androgen levels were not altered by LP diet. It is well-recognized that androgens are important neurodevelopmental factors (63). Androstenedione significantly alters the free fatty acid composition of the fetal brain (64). Excessive levels of androstenedione may induce cellular energy deficits and oxidative stress, potentially driving apoptosis (64). Thus, it might be hypothesized that the reduced androgen levels in the brain of LP males might serve to protect these animals from these adverse effects of IUGR [reviewed by Miller et al. (58)]. Brain compositional changes in free fatty acids warrant further research in our model. The stable androgen levels in the brain of female LP rats could be associated with their ability to metabolize androgens as precursors for estradiol (not determined in this study) (65). Beyond its impact on neurodevelopment, estradiol is known to exert anti-apoptotic effects (66). However, Fernandez-Twinn et al. (39) could only detect an end-gestational rise in circulating estradiol in LP dams of their protein restriction rat model, while no such effect was evident in the fetuses.

Fetal brain CRH levels were close to the lower limit of quantification via LC-MS/MS. Our finding that LP whole brains showed a trend to lower levels of CRH might, however, indicate alterations of superordinate control centers of the HPA-axis. As further targeted histomorphological assessment of these centers (67) is pending in our animal model, we hypothesize that the reduced levels of circulating corticosterone in LP whole blood are associated with reduced CRH in LP brain, despite the lack of significance. A study by Li et al. regarding HPA-axis function in nutritional IUGR of the baboon showed that CRH was the major releasing hormone driving ACTH and cortisol secretion in their model (68). In contrast to our results in the LP rat, the baboon (68), alike other models of maternal nutritional restriction (discussed below), showed an up-regulation of HPA-axis activity. We are currently unaware of other studies examining fetal CRH in the brain of LP rats.

### Effects of Low Protein Diet on Placental Hsd11b2 Expression and Function

LP diet led to a significantly reduced placental Cort/DH-Cort-ratio. The reduction of placental corticosterone levels appeared to be independent of organ-to-body weight ratio, similarly to whole blood. While Cort/DH-Cort-reduction did not reach significance in female LP placentas, the Cort/DH-Cort-ratio in whole blood of female LP rats was significantly reduced, alike in male littermates. The lack of significance in female LP placentas might be associated with the small group size. Thus, we concluded that LP diet did not induce fetal hypercortisolism in both sexes in our model.

There is a controversy (39) as to whether intrauterine glucocorticoid exposure is responsible for the reduction of birth weights in the LP model beyond the prevalent deprivation of amino acids (39, 69–71). Our own data does not support this notion. Steroid measurements of whole blood and tissue, via LC-MS/MS, might help to clarify this issue as direct evidence of altered levels of circulating glucocorticoids in the protein restriction model is scarce [ELISA, (39)] and largely based on reports on reduced mRNA expression levels of *Hsd11b2* as a surrogate for enzymatic activity (11, 12, 37, 38).

Jensen Peña et al. recently showed that chronic maternal restraint stress during gestational days 14–20 induced IUGR in Long Evans rats. They observed a significant decrease in placental *Hsd11b2* mRNA expression, associated with an increase in local *Hsd11b2* DNA methylation (40). Accordingly, fetal overexposure to maternal corticosterone was assumed to be involved in the pathogenesis of IUGR. However, no local or circulating glucocorticoid levels were determined in this study. Interestingly, while *Hsd11b2* mRNA levels in the brain remained unaffected (40), the DNA methylation patterns within the *Hsd11b2* promoter were similar to the ones determined in placenta, indicating a certain neuroplacental correlation (40).

In our model, we did not detect significant expressional changes of *Hsd11b1* and *2* mRNA in placenta. Our tissue LC-MS/MS method allows for the direct analysis of local steroid metabolism. Based on the observed dichotomy between our qRT-PCR and LC-MS/MS, we hypothesize that the latter method might be superior regarding the detection sensitivity of alterations in glucocorticoid metabolism. Interestingly, others have just recently found great variability in multiple mRNA species associated with cortisol production, metabolism, and action (72).

### Rat Models of Intrauterine Malnutrition and the Role of Glucocorticoids

The role of fetal overexposure to glucocorticoids in IUGR animal models of nutritional restriction remains somewhat unclear, partly due to the differences in feeding, and analytical methods used: employing a rat model where dams were kept on a restricted diet in late gestation (50% of their daily *ad libitum* intake), Blondeau et al. found increased maternal and fetal levels of corticosterone using radio-immunoassays (73). Fetuses had low birth weight and significantly reduced pancreatic insulin content. Thus, an inverse correlation of circulating maternal glucocorticoid levels with fetal β-cell mass was hypothesized (73). Similarly, other studies involving maternal undernutrition (either 50% or protein restriction) showed an increase in maternal levels of corticosterone and a reduction of placental *Hsd11b2* in rats (11, 13).

In contrast, Fernandez-Twinn et al. found serum corticosterone (ELISA detection) in both maternal and fetal circulation (39) to be unaffected by maternal protein restriction using a Wistar rat model of isoenergetic protein restriction throughout gestation ]80 g protein/kg vs. 200 g protein/kg (74), *ad libitum*]. However, they were able to identify endocrine changes in their dams (hyperglycemia with concomitant hyperinsulinism, increased levels of prolactin and decreased progesterone levels in early pregnancy; compensatory elevated oestradiol levels and reduced leptin levels in late pregnancy), that might contribute to the programming of poor glucose tolerance, insulin resistance, and the metabolic syndrome in their animals (39). By then, glucocorticoid levels had not been studied in the rat protein restriction model. Hence, based on these findings, Fernandez-Twinn et al. concluded that glucocorticoid levels may not have played a major role in direct programming of eventual adult disease in the developing fetus of their model of protein restriction (39).

Our model of protein restriction has not been characterized to develop postnatal disturbance of glucose tolerance or diabetes in young animals (75) and we have found a significant reduction of corticosterone in our LP rats. Thus, we hypothesize, that the hyperglycemic metabolic state of their dams (39) might have masked potentially reduced glucocorticoid levels so that corticosterone levels presumably matched those found in their NP controls. Exaggerated glucocorticoid secretion has been described to be present in diabetic animals and patients (76). Etiopathologically, the observed maternal hyperglycemia might have been due to the fact that their protein restriction model (74) mainly used monosaccharides to achieve isocaloric energy supplementation (39) and that *ad libitum* fed LP dams further showed an increased daily food intake (39). In contrast, such an effect did not occur in our model (daily food intake 25 g) and isocaloric conditions were fostered by increased substitution of polysaccharides and fat. Also, we observed an increase of the placenta-to-body weight ratio at E18.5, while Fernandez-Twinn et al. observed the ratio to be unchanged (E18), or rather reduced (E21) (39). This might suggest a higher placental capacity for the compensation of maternal protein restriction in our model.

In light of the presented results, one might hypothesize that the maternal food restriction model (i.e., 50% total intake restriction) might more closely resemble models of intrauterine stress than our model of protein restriction, as it leads to increased fetal glucocorticoids. Conclusions drawn from comparison of our study with the only other protein restriction model that examined fetal corticosterone levels (39) seem limited by the above-mentioned maternal metabolic differences. However, corticosterone levels of LP fetuses never exceeded NP levels in both studies. In line with this finding, our previous work (77) indicates that adult male LP rats did not show changes in their circulating glucocorticoid levels, postnatally.

### Potential Role of Progesterone in Energy Homeostasis

Progesterone is an essential neuroactive steroid during gestation (78). It is synthesized in maternal tissue, placenta, and the fetal brain, the latter of which is the major target of the steroid (79). Increased local biosynthesis of progesterone and its derivatives may contribute to adaptive processes in maternal and fetal brain during pregnancy (80). Progesterone also exerts neuroprotective functions (78).

While others found progesterone levels of protein restricted dams to be temporarily reduced, we did not detect an influence of LP diet on maternal and fetal progesterone levels at E18.5, despite a reduction in corticosterone and Cort/DH-Cort-ratio in placenta, fetal whole blood, and fetal brain. Thus, it might be speculated that the observed reduction of active glucocorticoids in our LP animals could have resulted, in part, from an organismal effort to maintain constant progesterone levels. Based on our findings, it can be hypothesized that glucocorticoid metabolism is reduced in favor of progesterone maintenance in our LP rats. Interestingly, progesterone is also known to enhance amino acid utilization by the liver (81, 82), which could be of relevance for energy homeostasis in our LP rats.

### Limitations

The presented results are limited by the small power of our study. Thus, some of our findings, especially of sex-related differences, represent trends and have to be interpreted with care. The expression of *Hsd11b 1* and *2* was determined on the mRNA level only, however, it remains elusive how mRNA expression translates to protein levels and enzymatic activity in target tissues. While LC-MS/MS has already served as a valid tool for the investigation of glucocorticoid turnover rates in tissue (9), we lacked sufficient amounts of material to perform microsomal enzymatic activity analyses in our study. Furthermore, as only a single time-point of gestation was examined, so far, dynamics in steroid metabolism during pregnancy remain undetermined. It needs to be noted, that the displayed results of our work are only focused on the analysis of fetuses from the proximal uterine horn, which represents the site with the highest maternal blood supply (13). Methodologically, we have avoided brain perfusion prior to LC-MS/MS tissue analysis, as blood contamination has been shown to have little effect on brain steroid levels and saline perfusion may significantly alter local steroid levels (47). Furthermore, we used anesthesia by isoflurane before sacrifice in all animals. While this technique avoids induction of glucocorticoids caused by the injection of anesthetic compounds (83), Bekhbat et al. have recently shown that the use of isoflurane might sex-specifically alter corticosterone levels as determined via ELISA (84). As we currently cannot rule out artifacts in response to collection paradigms, caution has to be used when transferring the presented data to models with different anesthesia.

## Conclusion

LP diet induced IUGR in our rats, yet it did not impair placental Hsd11b2 gatekeeping function in our study. We did not observe fetal hypercortisolism, as described by other rat models of IUGR (especially general undernutrition and stress models). In contrast, fetal corticosterone levels were significantly reduced in the LP group. This finding seemed independent of sex and was evident in whole blood, placenta and fetal brain. We hypothesize that our LP model does not represent a genuine model of intrauterine stress. Accordingly, our model might not be appropriate to study mechanisms involved in modulating fetal exposure to maternal steroids in IUGR. Thus, while the above-mentioned IUGR models imply that alterations of neuroplacental glucocorticoid cross-talk predispose for neurodevelopmental sequelae, this mechanism might not be present in our LP model. Our maternal protein restriction model may be seen as an equivalent to maternal protein malnutrition in developing countries (85) and might aid in understanding of mechanisms involved in fetal development under protein restriction conditions and the associated postnatal sequelae [especially reno-vascular and cardiac (3, 4, 46, 86)]. However, these may not be significant to and/or representative of mechanisms involved in intrauterine stress in general. At this time we cannot exclude that fetal hypercortisolism was preexistent at earlier gestational stages and that our analysis only determined a certain fatigue of HPA-axis function at the end of pregnancy. Nevertheless, the observed endocrine traits could suggest fetal efforts to achieve anabolic conditions, e.g., brain sparing, in the state of amino acid shortage. If such an effect was present in LP fetuses, it might negatively affect neurodevelopment. Our future studies will, therefore, include additional analysis of postnatal behavior, glucocorticoid levels, tissue metabolomics, and longitudinal steroid profiling beyond corticosterone, e.g., of progesterone derivatives.

## Data Availability

All relevant datasets for this study are included in the manuscript and the supplementary files. The raw data supporting the conclusions of this manuscript will be made available by the authors, without undue reservation, to any qualified researcher.

## Author Contributions

AH and FF designed the experiments and supervised the study. MS, RW, NC, and FF conducted and interpreted the experiments. MS, MCS, FF, and MRa performed the data analysis. MS, FF, and MRa wrote the manuscript. FF, MCS, and MS participated in the statistical analysis. WR, CM-C, AH, HH, MCS, and MRu critically revised the manuscript. All authors read and approved the final version of the manuscript.

### Conflict of Interest Statement

The authors declare that the research was conducted in the absence of any commercial or financial relationships that could be construed as a potential conflict of interest.
