# Supplementary material for: Influence of Low Protein Diet-Induced Fetal Growth Restriction on the Neuroplacental Corticosterone Axis in the Rat
Source: Front Endocrinol (Lausanne). 2019 Mar 11;10:124. doi: 10.3389/fendo.2019.00124 (PMC6421269; doi:10.3389/fendo.2019.00124)
Supplement: Supplementary file 1 [file Table_1.DOCX]

| **Hormones** | **Corticosterone** | **Dehydrocorticosterone** | **Progesterone** | **Testosterone** | **Deoxycorticosterone** |
| --- | --- | --- | --- | --- | --- |
| **Maternal** | 12.99 | 13.55 | 20.76 | 49.55 | 9.29 |
| **Fetal** | 18.34 | 11.61 | 12.93 | n/a | 16.21 |

**Supplementary Table S1:** Conversion factors of VAMS to EDTA-plasma.
