# Supplementary material for: Influence of Low Protein Diet-Induced Fetal Growth Restriction on the Neuroplacental Corticosterone Axis in the Rat
Source: Front Endocrinol (Lausanne). 2019 Mar 11;10:124. doi: 10.3389/fendo.2019.00124 (PMC6421269; doi:10.3389/fendo.2019.00124)
Supplement: Supplementary file 2 [file Table_2.docx]

**Supplementary Table S2:** Outliers not participating in the final analysis

| # | Steroid | Group | Tissue | Animal ID | Tissue level  (pmol/g) | Sex |
| --- | --- | --- | --- | --- | --- | --- |
| 1. | Corticosterone | NP | B | M7/F2 | 251.40 | female |
| 2. | Corticosterone | LP | B | M3/F5 | 278.86 | male |
| 3. | Corticosterone | LP | B | M3/F7 | 143.99 | male |
| 4. | Deoxycorticosterone | NP | B | M7/F1 | 38.22 | male |
| 5. | Deoxycorticosterone | LP | B | M5/F1 | 19.49 | female |
| 6. | Progesterone | NP | P | M9/F9 | 205.10 | female |

Legend: M = dam, F = fetus, B = brain, P = placenta, NP = normal protein (control),
LP = low protein (IUGR).
