# Supplementary material for: Influence of Low Protein Diet-Induced Fetal Growth Restriction on the Neuroplacental Corticosterone Axis in the Rat
Source: Front Endocrinol (Lausanne). 2019 Mar 11;10:124. doi: 10.3389/fendo.2019.00124 (PMC6421269; doi:10.3389/fendo.2019.00124)
Supplement: Supplementary file 3 [file Table_3.docx]

**Supplementary Table S3a**: Sex-specific steroid & CRH analysis of fetal brain.

|  | **Steroids in fetal brain** (pmol/g) | **NP** | **LP** | **p-value** |
| --- | --- | --- | --- | --- |
|  | **n** | 4 | 5 |  |
| **Male** | **Androstenedione** | 4.59 ± 0.61 | 2.73 ± 1.12 | 0.011^†^ |
|  | **Corticosterone (Cort)** | 97.40 ± 23.97 | 54.50 ± 7.00 | 0.001^†^ |
|  | **Dehydrocorticosterone (DH-Cort)** | 98.43 ± 35.65 | 114.90 ± 47.71 | ns^†^ |
|  | **Ratio (Cort/DH-Cort)** | 1.04 ± 0.15 | 0.57 ± 0.14 | <0.001^†^ |
|  | **Deoxycorticosterone** | 12.17 ± 3.66 | 8.33 ± 4.39 | ns^†^ |
|  | **Progesterone** | 49.34 ± 12.56 | 38.23 ± 24.00 | ns^†^ |
|  | **Testosterone** | 3.72 ± 0.97 | 1.94 ± 1.53 | 0.025^†^ |
|  | **CRH** | 1.30 ± 0.65 | 0.73 ± 0.29 | ns^†^ |
|  |  |  |  |  |
| **Female** | **Androstenedione** | 1.83 ± 0.43 | 1.98 ± 0.91 | ns^†^ |
|  | **Corticosterone (Cort)** | 99.30 ± 5.27 | 50.52 ± 14.96 | <0.001^†^ |
|  | **Dehydrocorticosterone (DH-Cort)** | 110.60 ± 22.87 | 127.20 ± 28.14 | ns^†^ |
|  | **Ratio (Cort/DH-Cort)** | 0.95 ± 0.16 | 0.40 ± 0.11 | <0.001^†^ |
|  | **Deoxycorticosterone (DOC)** | 13.73 ± 3.61 | 7.05 ± 2.72 | 0.032^†^ |
|  | **Progesterone** | 45.96 ± 7.72 | 38.04 ± 13.25 | ns^†^ |
|  | **Testosterone** | 0.01 ± 0.03 | 0.02 ± 0.04 | ns^†^ |
|  | **CRH** | 1.69 ± 1.12 | 0.64 ± 0.35 | ns^†^ |
| (†) Two-way Anova. | | | | |

**Supplementary Table S3b:** Sex-specific steroid analysis of placenta.

|  | **Steroids in placenta** (pmol/g) | **NP** | **LP** | **p-value** |
| --- | --- | --- | --- | --- |
|  | **n** | 4 | 5 |  |
| **Male** | **Androstenedione** | 9.10 ± 1.78 | 10.32 ± 2.32 | ns^†^ |
|  | **Corticosterone (Cort)** | 286.70 ± 37.08 | 164.10 ± 52.78 | 0.001^†^ |
|  | **Dehydrocorticosterone (DH-Cort)** | 302.10 ± 55.08 | 223.20 ± 69.82 | ns^†^ |
|  | **Ratio (Cort/DH-Cort)** | 1.02 ± 0.19 | 0.62 ± 0.27 | 0.019^†^ |
|  | **Deoxycorticosterone** | 17.09 ± 5.19 | 10.01 ± 3.93 | 0.031^†^ |
|  | **Progesterone** | 45.50 ± 16.16 | 49.60 ± 16.81 | ns^†^ |
|  | **Testosterone** | 1.22 ± 0.27 | 1.03 ± 0.21 | ns^†^ |
|  |  |  |  |  |
| **Female** | **Androstenedione** | 6.79 ± 1.85 | 6.93 ± 3.49 | ns^†^ |
|  | **Corticosterone (Cort)** | 259.00 ± 20.50 | 135.20 ± 42.35 | 0.001^†^ |
|  | **Dehydrocorticosterone (DH-Cort)** | 317.40 ± 49.99 | 245.90 ± 46.36 | ns^†^ |
|  | **Ratio (Cort/DH-Cort)** | 0.84 ± 0.13 | 0.56 ± 0.14 | ns (0.086) ^†^ |
|  | **Deoxycorticosterone (DOC)** | 18.04 ± 3.50 | 10.06 ± 2.56 | 0.016^†^ |
|  | **Progesterone** | 42.87 ± 10.77 | 42.40 ± 12.73 | ns^†^ |
|  | **Testosterone** | 0.96 ± 0.60 | 0.59 ± 0.49 | ns^†^ |
| (†) Two-way Anova. | | | | |
