# Supplementary material for: Influence of Low Protein Diet-Induced Fetal Growth Restriction on the Neuroplacental Corticosterone Axis in the Rat
Source: Front Endocrinol (Lausanne). 2019 Mar 11;10:124. doi: 10.3389/fendo.2019.00124 (PMC6421269; doi:10.3389/fendo.2019.00124)
Supplement: Supplementary file 4 [file Table_4.docx]

**Supplementary Table S4:** Steroid metabolism of progesterone in fetal whole blood, brain and placenta.

|  | **Ratio** | **NP** | **LP** | **p-value** |
| --- | --- | --- | --- | --- |
|  | **n** | 4 | 5 |  |
| **Whole blood** | **Progesterone/Corticosterone (Cort)** | 0.019 ± 0.005 | 0.035 ± 0.011 | 0.026^#^ |
|  | **Progesterone/Deoxycorticosterone (DOC)** | 0.47 ± 0.20 | 0.71 ± 0.22 | ns^#^ |
|  |  |  |  |  |
| **Brain** | **Progesterone/Corticosterone (Cort)** | 0.51 ± 0.12 | 0.84 ± 0.33 | ns^#^ |
|  | **Progesterone/Deoxycorticosterone (DOC)** | 3.84 ± 1.33 | 5.02 ± 0.83 | ns^#^ |
|  |  |  |  |  |
| **Placenta** | **Progesterone/Corticosterone (Cort)** | 0.17 ± 0.04 | 0.35 ± 0.17 | ns^#^ |
|  | **Progesterone/Deoxycorticosterone (DOC)** | 2.83 ± 0.65 | 4.91 ± 0.72 | 0.003^#^ |
| (#) Welch’s *t*-test. | | | | |
